# High-grade postoperative complications affect survival outcomes of patients with colorectal Cancer peritoneal metastases treated with Cytoreductive surgery and Hyperthermic Intraperitoneal chemotherapy

**DOI:** 10.1186/s12885-020-07756-7

**Published:** 2021-01-07

**Authors:** Sicheng Zhou, Qiang Feng, Jing Zhang, Haitao Zhou, Zheng Jiang, Zheng Liu, Zhaoxu Zheng, Haipeng Chen, Zheng Wang, Jianwei Liang, Wei Pei, Qian Liu, Zhixiang Zhou, Xishan Wang

**Affiliations:** 1 Department of Colorectal Surgery, National Cancer Center/National Clinical Research Center for Cancer/Cancer Hospital, Chinese Academy of Medical Sciences and Peking Union Medical College, Beijing, 100021 China; 2 Department of Abdominal Surgery, Huanxing Cancer Hospital, Beijing, 100122 China

**Keywords:** Peritoneal metastasis, Cytoreductive surgery, Hyperthermic intraperitoneal chemotherapy, Morbidity, Prognostic factors

## Abstract

**Background:**

This study aimed to evaluate the impact of postoperative complications on long-term survival in patients with peritoneal metastasis (PM) arising from colorectal cancer (CRC) treated with cytoreductive surgery (CRS) and hyperthermic intraperitoneal chemotherapy (HIPEC).

**Methods:**

Patients with PM arising from CRC treated with CRS and HIPEC were systematically reviewed at the China National Cancer Center and Huanxing Cancer Hospital from June 2017 to June 2019. High-grade complications that occurred within 30 days were defined as grade 3 to 4 events according to the Common Terminology Criteria for Adverse Events (CTCAE) classification. Univariate and multivariable Cox regression models for overall survival were created. Predictors of high-grade postoperative complications were evaluated with univariate and multivariate logistic regression analyses.

**Results:**

In all, 86 consecutive cases were included in this study. Forty-one patients (47.7%) developed postoperative complications, while 22 patients (25.6%) experienced high-grade complications. No mortality occurred during the postoperative period. The median survival of all patients was 25 months, and the estimated 3-year overall survival (OS) rate was 35.0%. In the multivariable Cox regression analysis, a high peritoneal carcinomatosis index (PCI) score (HR, 1.07, 95% CI, 1.01–1.14; *P*=0.015) and grade 3–4 postoperative complications (HR, 1.86, 95% CI, 1.22–3.51; *P*=0.044) correlated with worse overall survival. High estimated blood loss (OR, 1.01, 95% CI, 1.01–1.02; *P*< 0.001) was identified as an independent risk factor for developing high-grade complications.

**Conclusion:**

Careful patient selection, high levels of technical skill and improved perioperative management are crucial to ensure patient survival benefits after CRS+HIPEC.

## Background

Colorectal cancer (CRC) is the third most prevalent malignancy worldwide, with more than 1 million new cases in 2018 [1]. Peritoneal metastasis (PM) from colorectal cancer is a form of disease progression that affects approximately 10% of patients at the first surgery [2, 3] and is also an important reason for the poor prognosis of this disease [4]. Cytoreductive surgery (CRS) combined with hyperthermic intraperitoneal chemotherapy (HIPEC) has gradually become the standard treatment for PM arising from CRC after arduous exploration and practice [5–8]. For specifically selected patients, the median survival can reach approximately 40 months after CRS and HIPEC treatment for PM arising from CRC [9, 10].

Despite increasing evidence of the therapeutic efficacy of CRS+HIPEC, its safety has been doubted due to its high rates of morbidity and mortality. Recently, a large series composed of patients with varied tumour sources reported that the overall morbidity rate [11–18], severe morbidity rate [8, 13, 17, 19–26], and mortality rate [11, 17, 19, 20, 27, 28] after CRS+HIPEC were 37.9–60.5%, 6.4–35% and 2.5–4.6%, respectively. Moreover, it has been reported in the literature that the occurrence of postoperative complications after CRS+HIPEC is an independent negative prognostic factor affecting patients with PM arising from CRC [19]. Therefore, the aim of this study was to evaluate the perioperative safety and identify the risk factors for postoperative complications after CRS+HIPEC in our institution. Additionally, we sought to investigate the impact of postoperative complications on overall survival to guide future management.

## Methods

### Patient selection

After obtaining approval from the ethics committee of the Cancer Hospital, Chinese Academy of Medical Sciences (NCC 2017-YZ-026, Oct 17, 2017), all patients with synchronous or metachronous PM arising from CRC who underwent CRS with HIPEC were retrospectively collected between June 2017 and June 2019 at the National Cancer Center and Huanxing Cancer Hospital. The inclusion criteria included the following: (1) pathologically diagnosed malignant colorectal tumour; (2) age between 18 and 75 years; and (3) Eastern Cooperative Group (ECOG) score ≤ 1. (4) patients performed three times complete HIPEC procedure. The exclusion criteria were as follows: (1) a previous history of other cancers; (2) patients undergoing palliative surgery, such as bypass surgery or simple ostomy; (3) peripheral blood neutrophil count <2000 × 10^9^/L or platelet count <100 × 10^9^/L; (4) abnormal liver function: serum total bilirubin (TBIL) level > 21 μmol/L or alanine transaminase (ALT) level > 40 U/L; and (5) abnormal renal function: serum creatinine level > 106 μmol/L or urea level > 7.1 mmol/L. Finally, 86 patients met the above criteria and were included in this study. All enrolled patients in this study provided written informed consent.

### Perioperative management

Patients underwent routine preoperative evaluations to assess their general condition, calculate the peritoneal carcinomatosis index (PCI), and identify distant metastasis, including laboratory examinations, abdominal contrast-enhanced computed tomography (CT), and pelvic magnetic resonance imaging. All potential CRS+HIPEC cases were discussed in multidisciplinary team (MDT) meetings that incorporated radiologists and medical and surgical oncologists to develop a consensus for a comprehensive treatment strategy. The PCI was used to assess the degree of PM and has scores from 0 to 3 for each of the 13 defined areas of the abdominal cavity [29, 30]. Toxicity indexes of chemotherapy (blood, liver, and kidney toxicity), including neutrophil count, platelet count, ALT level, TBIL level, creatinine level and urea level, were measured in the morning on postoperative days (PODs)1, 3, and 5. Patients with severe liver and kidney impairment or myelosuppression will be discontinued from additional HIPEC procedure and exclude form the study.

### Operative details

Depending on the location of the PM, CRS consists of various surgical and peritonectomy procedures, including pelvic peritonectomy, anterior peritonectomy, omentectomy, ovariectomy, and hysterectomy following the Sugarbaker techniques [31]. The completeness of the cytoreduction score (CC score) was recorded at the end of each operation. CC-0/1 was deemed complete cytoreduction, with CC-2/3 considered incomplete cytoreduction [32]. Incomplete cytoreduction is the removal of as much as is visible to the naked eye, but there is bound to be residual tumor. Patients undergoing palliative surgery, such as bypass surgery or simple ostomy, are not included. All the 14 patients with liver metastasis were isolated and resectable and they underwent simultaneous resection of liver metastasis after CRS. After resection, three outflow drainage tubes and one inflow drainage tube were routinely placed in the abdomen to prepare for HIPEC. There were two chemotherapeutic regimens during HIPEC in this study. Oxaliplatin (200 mg/m^2^) and raltitrexed (3 mg/m^2^) with or without lobaplatin (50 mg/m^2^) were used for intraperitoneal chemotherapy. After catheterization was completed, patients in both groups were treated with a mixed solution of chemotherapy agents and 3 L of saline solution in the abdominal and pelvic cavity for 60 min at 42–43 °C. After that, four catheters remain in original position, and two more HIPEC procedures with same chemotherapeutic regimens as well as perfusion time were performed again on the second and fourth days after surgery in the ward. Two surgical specialists with more than 20 years of experience in gastrointestinal surgery performed operations at the two central, while the exactly similar HIPEC technique and postoperative treatment were performed at both centers.

### Postoperative complications

Postoperative complications that occurred within 30 days were recorded and graded in accordance with the Common Terminology Criteria for Adverse Events (CTCAE) classification [33]. High-grade complications were described as grade 3 and 4 events. Leukopenia was graded as follows: grade III: < 2000–1000/mm3, and grade IV: < 1000/mm3. Neutropenia was graded as follows: grade III: < 1000–500/mm3, and grade IV: < 500/mm3. Thrombocytopenia was graded as follows: grade III: < 50,000–25,000/mm3, and grade IV: < 25,000/mm3.

### Follow-up

The postoperative systemic chemotherapy regimen was developed by two medical oncologists with expertise in the gastrointestinal field. All patients were scheduled to receive follow-up through outpatient visits or telephone interviews every 3 months for the first two years and then every 6–12 months for the next 3 years until death due to recurrence and metastasis or July 31, 2020, whichever came first. Routine examinations included CT imaging of the thorax, abdomen and pelvis and blood tests of tumour markers when appropriate. The long-term endpoint of this study was 3-year overall survival (OS).

### Statistical analysis

SPSS 24.0 software (IBM, Armonk, NY, USA) was used to for statistical analysis. All results are expressed as the median with an interquartile range for continuous variables and absolute and percentage frequencies for categorical variables. Survival analysis was calculated using the Kaplan–Meier method, and data were analysed using the log rank test. All significant univariate variables were applied in the multivariate Cox regression model, and their independent prognostic value was evaluated. Univariate logistic regression models using low-grade complications and the absence of complications as references were performed to identify independent risk factors for developing high-grade complications. Variables with *P*< 0.05 in univariate analysis were included in the multivariate logistic regression model. A *P* value < 0.05 was considered statistically significant.

## Results

### Patient demographics and clinical characteristics

The demographical and clinical characteristics of patients are summarized in Table 1. A total of 86 consecutive patients whose average age was 55.6 ± 11.7 (range, 27–74) years were included in our study, including 45 (52.3%) male and 41 (47.7%) female patients. Among these patients, 52 (60.5%) exhibited synchronous PM, and 41 (51.2%) underwent preoperative chemotherapy. The most common primary tumour types and histology observed were colon cancer (73.3%) and adenocarcinoma (64.0%), respectively. The average preoperative CEA and CA19–9 levels were 33.4 ± 65.2 (range, 0.6–291.7) and 68.3 ± 87.7 (range, 0.7–363.5), respectively.

**Table 1:** Patient demographics of 86 patients underwent CRS+HIPEC

Characteristics	Total (*n*=86)
Age at operation (y, mean ± SD) (range)	55.6 ± 11.7 (27–74)
Gender (%)	
Male	45 (52.3)
Female	41 (47.7)
Preoperative chemotherapy (%)	41 (51.2)
Presentation of PM (%)	
Synchronous	52 (60.5)
Metachronous	34 (39.5)
Site of original (%)	
Colon	63 (73.3)
Rectum	23 (26.7)
Preoperative CEA level (ng, mean ± SD) (range)	33.4 ± 65.2 (0.6–291.7)
Preoperative CA19–9 level (ng, mean ± SD) (range)	68.3 ± 87.7 (0.7–363.5)
Histology (%)	
Adenocarcinoma	55 (64.0)
Mucinous	31 (36.0)
Liver metastases	17 (19.8)
HIPEC regimen	5 (6.6)
Lobaplatin+Oxaliplatin+Raltitrexed	40 (46.5)
Oxaliplatin+Raltitrexed	46 (53.5)
PCI score	11.2 ± 5.7 (2–24)
Operation time (min, mean ± SD) (range)	264.0 ± 70.3 (145–510)
Estimated blood loss (ml, mean ± SD) (range)	121.4 ± 107.8 (20–500)
Presence of ascites	37 (43.0)
CC score	
CC 0–1	54 (62.8)
CC 2–3	32 (37.2)
Postoperative Complications (grade 1–4) (%)	41 (47.7)
Postoperative Complications (grade 3–4) (%)	22 (25.6)
Mortality (%)	0 (0)
Total hospital stay (day, mean ± SD) (range)	15.4 ± 6.0 (7–44)

HIPEC with lobaplatin, oxaliplatin, and raltitrexed was used in 40 (46.5%) cases, and HIPEC with oxaliplatin and raltitrexed was used in the remainder of the patients (53.5%). Patients had a median PCI score of 11.2 ± 5.7 (range 2–24), and 62.8% of them underwent complete cytoreduction (CC score 0–1). The average operative time was 244.0 ± 70.3 (range, 145–510). During the operation, patients had an estimated average blood loss of 121.4 ± 107.8 mL (range 20–500).

Following the operation and within 30 days, 41 patients (47.7%) developed postoperative complications, while 22 patients (25.6%) experienced high-grade complications (Table 2). No mortality occurred during the postoperative period. The most common complication observed was ileus (14.0%, *n* = 12), followed by abdominal abscess (10.5%, *n* = 9) and wound infection (9.3%, *n* = 8).

**Table 2:** Postoperative complications of 86 patients after CRS+HIPEC

Complications	Grade 1–2 complications		Grade 3–4 complications		All complications	
	n	%	n	%	n	%
Total	19	22.1	22	25.6	41	47.7
Blood disorders (grade 3–4)						
Leukopenia	2	2.3	0	0	2	2.3
Neutropenia	2	2.3	0	0	2	2.3
Thrombocytopenia	5	5.8	0	0	5	5.8
Cardiac disorders						
Arrhythmia	4	4.7	1	1.2	5	5.8
Respiratory disorder						
Pneumonia	4	4.7	2	2.3	6	7.0
Pleural efusion	2	2.3	2	2.3	4	4.6
Gastrointestinal disorders						
Anastomotic leakage	3	3.5	3	3.5	6	7.0
Ileus	6	7.0	6	7.0	12	14.0
GastrointestinaI hemorrhage	2	2.3	2	2.3	4	4.7
Renal and urinary disorders						
Urinary infection	2	2.3	0	0	2	2.3
Renal failure	0	0	1	1.2	1	1.2
Urinary retention	0	0	2	2.3	2	2.3
Other disorders						
Abdominal abscess	3	3.5	6	7.0	9	10.5
Rectovaginal fistula	0	0	1	1.2	1	1.2
Intra-abdominal hemorrhage	1	1.2	3	3.5	4	4.7
Wound infection	5	5.8	3	3.5	8	9.3

### Survival analyses

**Fig. 1:**
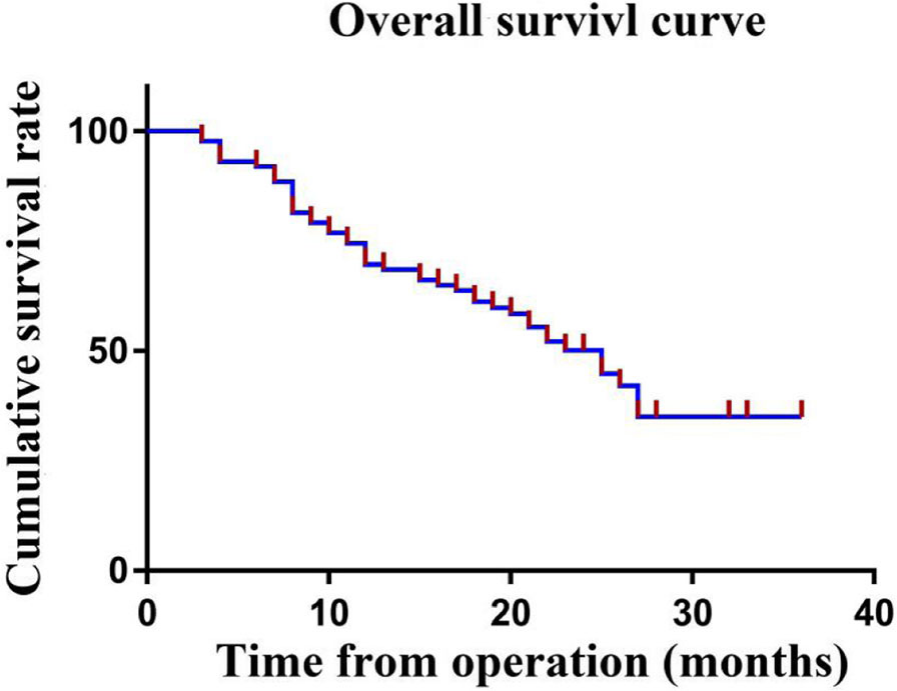
Overall survival rate of 86 patients after CRS+HIPEC

The median follow-up period was 19.5 (range, 3–36) months. The median survival for all patients was 25 months, and the estimated 1-, 2- and 3-year OS rates for the entire cohort were 69.7, 50.1, and 35.0%, respectively (Fig. 1). Cox univariate regression analysis identified the site of origin, PCI score, CC score, and grade 3–4 postoperative complications as factors associated with OS (Table 3). It is noteworthy that that the prognosis of patients with mucinous adenocarcinoma was worse than that of patients with non-mucinous adenocarcinoma, however, there was no statistical difference (*P*=0.096). In the multivariable Cox regression analysis, two variables emerged as independent prognostic factors: PCI score and grade 3–4 postoperative complications. Patients with high PCI scores (HR, 1.07, 95% CI, 1.01–1.14; *P*=0.015) and grade 3–4 postoperative complications (HR, 1.86, 95% CI, 1.22–3.51; *P*=0.044) had significantly worse overall survival (Table 3).

**Table 3:** Univariate and multivariate Cox regression analysis of overall survival in 86 patients after CRS+HIPEC

Variables	Overall survival			
	Univariate analysis		Multivariate analysis	
	HR(95%CI)	*P*	HR(95%CI)	*P*
Gender: male/female	1.21 (0.68–2.18)	0.517		
Age at operation	1.01 (0.99–1.04)	0.400		
Preoperative chemotherapy (no/yes)	0.79 (0.40–1.55)	0.489		
Synchronous/metachronous	0.80 (0.44–1.47)	0.476		
Site of original (colon/rectum)	2.13 (1.16–3.92)	0.015	1.71 (0.913–3.20)	0.094
Histology (adenocarcinoma/mucinous)	1.65 (0.92–3.00)	0.096		
Preoperative CEA level	1.00 (0.99–1.01)	0.161		
Preoperative CA19–9 level	1.00 (0.99–1.01)	0.342		
Liver metastases (no/yes)	1.36 (0.67–2.74)	0.397		
HIPEC regimen (lobaplatin/non-lobaplatin)	1.60 (0.87–2.95)	0.134		
Presence of ascites (no/yes)	1.34 (0.74–2.40)	0.335		
PCI score	1.01 (1.05–1.15)	< 0.001	1.07 (1.01–1.14)	0.015
CC score (0–1/2–3)	3.36 (1.85–6.08)	< 0.001	1.92 (0.96–3.83)	0.064
Grade 3–4 Postoperative complication (no/yes)	2.71 (1.48–4.94)	0.001	1.86 (1.22–3.51)	0.044
Leukopenia (no/yes)	1.31 (0.61–2.83)	0.487		
Neutropenia (no/yes)	1.31 (0.47–3.70)	0.605		
Thrombocytopenia (no/yes)	1.45 (0.80–2.66)	0.224		

### Factors for the development of high-grade complications

Factors associated with high-grade complications were determined with multinomial logistic regression and are summarized in Table 4. Univariate analysis showed that high estimated blood loss (*P<* 0.001) and high PCI score (*P*=0.008) were associated with an increased possibility of high-grade complications. However, other factors, including age, sex, American Society of Anesthesiologists (ASA) score, body mass index (BMI), preoperative chemotherapy, site of origin, liver metastases, HIPEC regimen, operative time, presence of ascites, and CC score, were not significantly associated with high-grade complications. Multivariate analysis showed that high estimated blood loss (OR, 1.41, 95% CI, 1.13–1.82; *P*< 0.001) was identified as an independent risk factor for developing high-grade complications.

**Table 4:** Predictive factors of 3–4 grade postoperative complications assessed by univariate and multivariate logistic regression analyses

Variables	Grade 3–4 postoperative complications			
	Univariate analysis		Multivariate analysis	
	OR(95%CI)	*P*	OR(95%CI)	*P*
Gender: male/female	1.45 (0.55–3.83)	0.456		
Age at operation	1.02 (0.98–1.07)	0.347		
ASA score (I-II/III-IV)	1.47 (0.48–4.50)	0.499		
Body mass index	1.08 (0.90–1.28)	0.342		
Preoperative chemotherapy (no/yes)	1.76 (0.52–5.92)	0.360		
Site of original (colon/rectum)	2.47 (0.88–7.0)	0.087		
Liver metastases (no/yes)	1.28 (0.40–4.14)	0.687		
HIPEC regimen (lobaplatin/non-lobaplatin)	0.48 (0.17–1.40)	0.177		
Operative time	1.01 (0.99–1.01)	0.164		
Estimated blood loss	1.62 (1.21–2.04)	< 0.001	1.41 (1.13–1.82)	< 0.001
Presence of ascites (no/yes)	1.14 (0.43–3.03)	0.790		
PCI score	1.12 (1.03–1.20)	0.008	1.22 (0.95–1.44)	0.085
CC score (0–1/2–3)	0.76 (0.28–2.05)	0.582		

## Discussion

Great progress in the treatment of PM from colorectal cancer has been made in recent years. The peritoneal surface is usually a common site of tumour dissemination from CRC, and an increasing number of studies support surgical CRS+HIPEC as a standard treatment option for PM [5–8]. Our findings demonstrate an overall median survival of 25 months and a 3-year survival rate of 35.0% for the entire cohort; these results are inferior to previous reports in the literature [19–23]. This may be due to the high proportion (37.2%) of patients who underwent incomplete cytoreduction and the presence of liver metastasis in 18.2% of patients in the present study.

It is essential to determine the selection factors that predict survival in PM patients undergoing this complex and potentially life-threatening procedure. However, given the lack of high-quality randomized data, the transition of CRS+HIPEC from experimental therapy to a standard treatment mode is still in progress. Until now, lessons learned from large-scale multi-institutional registry studies were that high PCI score, incomplete cytoreduction and severe carcinomatosis were associated with poor survival, even after CRS+HIPEC [27, 28]. Furthermore, Faron et al. [11] documented a linear correlation between PCI and overall survival. Woeste et al. [16] also found that both the preoperative PCI score and preoperative response to chemotherapy, together with the postoperative PCI score, were independent prognostic factors for overall survival. The present study confirmed that patients with high PCI scores had significantly worse overall survival (HR, 1.07, 95% CI, 1.01–1.14; *P*=0.015), which is similar to the above reports.

More attention has been given to the morbidity and mortality associated with the CRS+HIPEC procedure than to its efficacy. A recent systematic review by Chua et al. showed a morbidity range from 12 to 52% and a mortality range of 0.9–5.8% in 10 specified international treatment centres that were regarded as high-volume and experienced centres [34]. Our institution demonstrated that the overall morbidity rate, severe morbidity rate, and mortality rate after CRS+HIPEC were 47.7, 25.6% and 0, respectively, which is basically consistent with the results reported by international centres. The perioperative results reported by various institutions are quite different, which may be due to differences in diagnosis and treatment experience and cytoreductive techniques in various regions around the world. Furthermore, this difference is also attributed to the lack of unified standards for treatment parameters such as dose, temperature, perfusion time, and chemotherapy regimen in various institutions.

In addition, the present study showed that patients with high-grade complications had a worse prognosis than those without complications (HR, 1.86, 95% CI, 1.22–3.51; *P*=0.044). The most frequent complication observed in our patient cohort was ileus (14.0%), followed by abdominal abscess (10.5%). Tan et al. reported that patients without complications had significantly better survival than those with high-grade complications (HR, 0.35, 95% CI, 0.15–0.81; *P*< 0.001) [35]. A meta-analysis study including 717 patients who underwent CRS+HIPEC conducted by Narasimha et al. also showed that the presence of grade III/IV morbidity was independently associated with worse overall survival (HR, 1.59, 95% CI, 1.17–2.16; *P*=0.003) [36]. The above research supports our results. The occurrence of severe complications may worsen general conditions and hamper subsequent adjuvant therapies or treatment for recurrence. Therefore, improving surgical techniques and selecting suitable patients are crucial to exploiting all the benefits of this treatment strategy. The learning curve of this complex surgical procedure requires rigorous training, and a recent study suggested that surgical complications could be reduced as experience increased within an acceptable range [19].

The present study also assessed independent risk factors for the development of high-grade complications in patients undergoing CRS/HIPEC. Patients with high intraoperative blood loss were more likely to develop high-grade complications (HR, 1.41, 95% CI, 1.13–1.82, *P*< 0.001), probably because operative blood loss can reflect the complexity of the operation and the surgical risk. Therefore, preoperative imaging examinations and laparoscopic surgical exploration that can identify extensive disease and complex cytoreduction are necessary to assess the surgical risk. Likewise, Tan et al. demonstrated that under the premise of adjusting a multitude of factors, intraoperative blood loss remained associated with the development of complications [35]. Similarly, Macri et al. proved that the number of blood transfusions was an independent risk factor for severe postoperative complications [37]. Therefore, for patients with high operative risks and poor surgical tolerance, CRS+HIPEC needs to be performed prudently, and other adjuvant therapies that can replace this complex procedure can be tried.

The most significant limitation of our study is its retrospective nature, and only 86 patients were included, which may have caused some inherent selection bias. However, all therapeutic schedules of these patients were made by a multidisciplinary team specializing in colorectal cancer in our institution, and all included patient data were unabridged and reliable. Therefore, we believe that the results are reliable. Multicentre, large-scale, prospective studies are worth looking into to verify our results.

## Conclusion

On the basis of our retrospective analysis, the long-term survival outcomes were influenced by high PCI scores and high-grade postoperative complications after CRS+HIPEC in patients with PM of a CRC origin. In addition, intraoperative blood loss was an independent risk factor for the development of grade 3–4 postoperative complications. Careful patient selection, high levels of technical skill and improved perioperative management are crucial to ensure patient survival benefits after CRS+HIPEC.

## Data Availability

The datasets generated and/or analysed during the current study are not publicly available due to the data is confidential patient data but are available from the corresponding author on reasonable request.

## Abbreviations

PM: Peritoneal metastasis
CRC: Colorectal cancer
CRS: Cytoreductive surgery
HIPEC: Hyperthermic intraperitoneal chemotherapy
CTCAE: Common Terminology Criteria for Adverse Events
ECOG: Eastern Cooperative Group
TBIL: Total bilirubin
ALT: Alanine transaminase
PCI: Peritoneal carcinomatosis index
CT: Computed tomography
MDT: Multidisciplinary team
CC score: Completeness of the cytoreduction score
OS: Overall survival
